# Impact of oral conditions on salivary biochemical parameters in individuals with substance use disorder: a cross-sectional study

**DOI:** 10.1590/1807-3107bor-2025.vol39.053

**Published:** 2025-05-12

**Authors:** Julia Arruda BATISTA, Bruno WAKAYAMA, Rayara Nogueira de FREITAS, Gabriela Alice FIAIS, Antonio Hernandes CHAVES-NETO, Tânia Adas SALIBA, Artênio José Isper GARBIN, Clea Adas Saliba GARBIN

**Affiliations:** (a) Universidade Estadual Paulista – Unesp, School of Dentistry of Araçatuba, Department of Preventive and Restorative Dentistry, Araçatuba, SP, Brazil.; (b) Universidade Estadual Paulista – Unesp, School of Dentistry of Araçatuba, , Department of Basic Sciences, Araçatuba, SP, Brazil.; (c) Universidade Estadual Paulista – Unesp, School of Dentistry of Araçatuba, Department of Basic Sciences, Araçatuba, SP, Brazil.

**Keywords:** Oral health, Saliva, Public Health, Biomarkers

## Abstract

This study aimed to investigate oral conditions and their impact on salivary biochemical parameters in institutionalized individuals with and without substance use disorder. This was an epidemiological, cross-sectional, and clinical study. It included two groups, institutionalized individuals with substance use disorder (SUD group) and without substance use disorder (control group), each consisting of 60 participants. Salivary samples were analyzed for various parameters, while oral conditions were assessed using the DMFT index, community periodontal index, clinical attachment loss index, and need for prosthesis. Statistical analysis included the Mann-Whitney U test, t-tests, and correlation analysis (p ≤ 0.050). The SUD group showed a higher need for dental prostheses (p < 0.001) and more pronounced dental erosion (p < 0.001). This group also exhibited elevated DMFT indices, with significant associations in sextants with calculus (p = 0.010), periodontal pockets (p < 0.001), and attachment loss of 12 mm or more (p = 0.036). Regarding salivary parameters, the SUD group had high cortisol levels and significant correlations between uric acid and bleeding sextants (p = 0.024), salivary amylase and decayed teeth (p = 0.002), cortisol and the DMFT index (p = 0.045), and cortisol and the absence of DMFT (p = 0.042). In conclusion, individuals in the SUD group exhibited worse oral conditions than did those in the control group, suggesting a relationship between drug addiction and increased cortisol, uric acid, and salivary amylase levels.

## Introduction

Substance abuse has become a global concern^1,2^ that has been shaped by historical and cultural permissiveness and collective representation, having an impact on various social determinants.^1^According to health organizations, the use of psychoactive drugs has increased in the last decade, with cannabis, cocaine, and crack cocaine as the most frequently consumed substances.^2,3^ It is therefore reasonable to discuss this public health issue, given its traumatic individual repercussions, incitement to drug trafficking, violence, stigma, marginalization, and addiction.^4,5^

Drug addiction is a chronic relapsing disorder characterized by a complex multifactorial etiology, which can be defined as a lack of impulse control in response to exposure to psychoactive drugs. Although drug addiction is strongly associated with the voluntary desire to use substances, recent studies have revealed that immoderate use can cause damage to the serotonin and dopamine pathways.^6^ Psychoactive substances change the cognitive capacity of individuals with substance use disorder, leading to mood swings, risky behavior, and self-inflicted injury due to difficult information processing, impaired concentration, loss of decision-making skills, and reversal of executive functions.^7^ Thus, the lifestyle of these individuals not only disrupts central nervous system functions and the neuroaxis but also has consequences for their oral health.^8-10^

Some of the most prevalent diseases and conditions observed in this population include reduced salivary flow, halitosis, dental caries, periodontal disease, dental pain, dental erosion, edentulism, bruxism, changes in salivary pH, and oral lesions.^8,9,11^ These conditions can be aggravated by drug addiction, contributing to low self-esteem, poor self-care, and a reduced likelihood to seek dental care.^8^

In addition, the use of certain psychoactive substances can interfere with the amount and quality of salivary compounds, disrupting their balance and stability, and leading to the emergence and worsening of oral and dental morbidities and alterations.^10,12^ Salivary fluid contains biomarkers, which are immunoreactive compounds associated with the detection, risk assessment, prognosis, and monitoring of individuals’ health conditions.^12,13^ The easy collection of saliva, coupled with the non-invasiveness and cost-effectiveness of the procedure, has prompted recent studies to increasingly focus on identifying biochemical parameters, underscoring the growing importance of molecular diagnostics in addressing oral cavity pathologies.^12-14^

As psychoactive substances affect health status, it is essential to conduct clinical and laboratory monitoring of the oral health of individuals with substance use disorder to strengthen health actions and services designed to manage pathologies and promote health. Therefore, the objective of this study was to investigate oral conditions and their impact on salivary biochemical parameters in institutionalized individuals with and without substance use disorder.

## Methods

### Ethical aspects

This study was approved by the Research Ethics Committee (process no. CAAE: 57639722.9.0000.5420) and complied with all the ethical principles for conducting research involving human subjects, as required by Resolution No. 466/2012 of the Brazilian National Health Council, the Declaration of Helsinki, and the Nuremberg Code. The participants were informed about the study and signed the informed consent form (ICF).

### Study design

This is an epidemiological, cross-sectional, and clinical study conducted with institutionalized individuals with substance use disorder (SUD group) and without substance use disorder (control group). The study was carried out in two institutions located in the northwestern region of the state of São Paulo, Brazil, which had similar therapeutic and structural characteristics, as well as similar sociodemographic and educational profiles.^15^

This study followed the Strengthening the Reporting of Observational Studies in Epidemiology (STROBE) guidelines for cross-sectional studies.

### Sampling universe

To establish the sample universe, we searched the national and international literature to estimate the number of individuals with substance use disorder and control group participants from previous studies who share epidemiological aspects similar to those planned for the present investigation. Thus, we determined that the sample should include at least 120 individuals, with 60 participants in the SUD group and 60 in the control group.

The SUD group included 60 male individuals, aged 18 or older, from both institutions, all of whom were present during the data collection period and agreed to participate in the study. The inclusion criteria for the SUD group were as follows: chronic use of licit and illicit substances, absence of salivary gland diseases and/or lesions, and no spontaneous oral bleeding. Individuals were excluded from the study based on the following criteria: lack of mental clarity to complete the survey, people with disabilities, and individuals with insufficient salivary volume deemed inadequate for the analysis of salivary compounds.

The control group included 60 male individuals matched for age and sociodemographic characteristics who had no history of psychoactive substance use. The control group was subjected to the same exclusion criteria as the SUD group. Participant selection was parameterized in two social care institutions, following the inclusion and exclusion criteria and ensuring compatibility with the characteristics of the SUD group.

### Data collection

Data were collected in two stages (Figure). To identify participants’ characteristics, we employed interrogative methods using both objective and open-ended questions exclusively developed for this study after review of the relevant literature. We could then understand the sociodemographic characteristics of the substance users, assess their health from their perspective, and evaluate their oral and salivary conditions.

**Figure:**
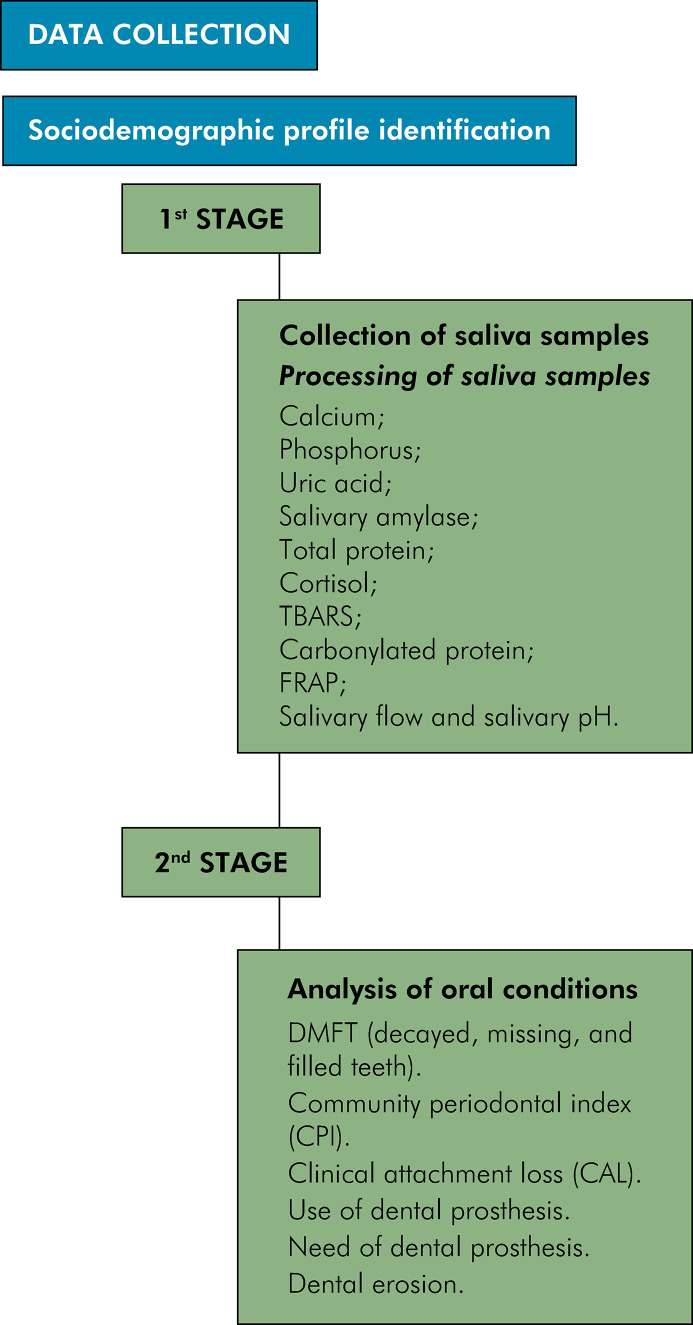
Study flowchart

### First stage — Collection of saliva samples

Saliva samples were collected following scheduled appointments with therapeutic institutions, requesting that the samples be collected between 8 a.m. and 10 a.m. to minimize the effects of the circadian rhythm.^15^ Study participants were instructed not to consume any type of drink or food one hour before the start of the study in order to avoid the risk of sample contamination. The saliva produced in the initial minute was discarded. In the following 10 minutes, the participants expectorated their saliva into a sterile 15 mL Falcon tube. The samples were then centrifuged for 10 minutes at 4ºC, separated into nine aliquots, and stored at -80ºC.

The reagents for the biochemical tests were supplied by Sigma-Aldrich (Germany/USA), and absorbance was measured on a microplate reader spectrophotometer (PowerWave 340, BioTek, Santa Clara , USA). The tests were conducted in duplicate and standardized based on the concentration of total proteins (TP), enabling the assessment of proportional differences in the biochemical analytes found in saliva. TP concentrations were determined by the Hartree-Lowry method (1972) and expressed in mg/L.

Participants’ salivary flow rate was determined using a standard density of 1.0 for total saliva. The rate (mL/min) was calculated by dividing the difference between the weights of the flask before and after collection by the collection time. Salivary pH was measured using 2 mL of saliva with the aid of pH-Fix 0-14 colorimetric strips (Macherey Nagel GmbH & Co. KG, Duren, Germany). The levels of calcium, phosphorus, and uric acid were quantified with Bioclin® colorimetric kits (Quibasa Química Básica Ltda., Belo Horizonte, Brazil), employing the cresolphthalein complexone method. Amylase (AMI) activity, using the commercial Labtest kit, according to the manufacturer’s instructions. The analysis of cortisol concentrations followed the same procedures for saliva collection described above. The samples were placed in 2 mL Eppendorf® (Sigma-Aldrich, Germany/USA) microtubes and then centrifuged at 100xg for four minutes, allowing cortisol concentrations to be analyzed in an Enzyme Linked Immuno Sorbent Assay (ELISA) using the ELK-Biotechnology commercial kit. Carbonylated protein (CP) was quantified using the alkaline method (DNPH), and lipid peroxidation was assessed by the analysis of the concentration of thiobarbituric acid reactive substances (TBARS). The total salivary antioxidant capacity was determined spectrophotometrically using the ferric reducing antioxidant power (FRAP) method. The results were calculated using a standard curve with various concentrations of FeSO4 solutions.

### Second stage — Analysis of oral conditions

The analysis of intraoral conditions followed the methodologies described in the fifth edition of Oral Health Surveys: Basic Methods,^16^ published by the World Health Organization (WHO), and the Manual for the Examiner and Annotator (2010) from the SB Brasil project.^17^ All the procedures for conducting an epidemiological survey were followed in strict compliance with biosafety standards. Oral examinations were conducted in an open environment, with the strict necessity of natural light for the visualization of the oral cavity.

Before the study, two researchers (examiner and recorder) conducted a calibration process with individuals of the same socioeducational profile and age range as those of the research population. The examined individuals were not included in the final sample. Before the fieldwork, the team was trained in conducting epidemiological studies, and intra-examiner calibration for the oral examinations yielded a kappa statistic of 0.93. The intraoral examinations were performed exclusively by the main researcher, while the completion and organization of forms, materials, and instruments were handled by the recorder.

Oral examinations were carried out using a wooden spatula, a clinical mirror, and a WHO probe to investigate caries experience through the DMFT (decayed, missing, and filled permanent teeth) index; periodontal disease using the community periodontal index (CPI) and the clinical attachment loss (CAL) index; use and need for dental prosthesis; and dental erosion. We employed CPI to assess health status, bleeding, and presence of calculus and/or periodontal pockets. The buccal, palatal and/or lingual surfaces were examined, focusing on the mesial, middle, and distal sections of the reference index teeth: 17, 16, 11, 26, 27, 37, 36, 31, 46, and 47. Among the 10 tooth elements analyzed, markings were made for six teeth, considering one per sextant and reporting the worst evaluated situation. Sextants with fewer than two remaining teeth were excluded. The CAL index is a complementary examination to CPI, which allows for the assessment of the periodontal attachment condition based on the cementoenamel junction. The same index teeth evaluated in the CPI, as well as the analyzed sites, were considered for this examination. During the evaluation of sextants, the worst condition presented by the CPI index may not correspond to the worst condition observed in the CAL. For the examinations, we employed the WHO periodontal probe to measure CAL, following the standardization and recommendations for oral epidemiological surveys. Additionally, the results were categorized according to the codes and criteria established by the examiner’s manual of the SB Brasil Project, 2010.^17^

### Statistical analysis

Descriptive statistics were used for the initial analysis of the results. The methodological goal was to summarize the variations in the numerical values found in the study, allowing for a comprehensive and structured understanding of the phenomenon. Bivariate analyses were employed to assess categorical (dependent and independent) variables, using either Pearson’s chi-square test or the likelihood ratio test with a significance level of p ≤ 0.050. The Kolmogorov-Smirnov test was applied before the comparative analysis of metric variables (dependent). We tested the hypothesis that the data follow a normal distribution. This hypothesis was rejected when the p-value was lower than 0.050, indicating that the data were not normally distributed. Either the t-test for means (parametric test) or the Mann-Whitney U test (non-parametric test) was applied in the analyses.

The correlation analysis (ρ) provided the associations between oral conditions and biochemical parameters, with the degree of relationship between the variables expressed by a coefficient that indicates simultaneous variation of the variables.

The data were tabulated using Excel for Windows 2010, and the statistical analysis was performed using Statistical Package for the Social Sciences (SPSS for Windows; version 20.0, Chicago, USA).

## Results

This study included 120 participants, 60 volunteers for the SUD group and 60 for the control group. Most participants were white (62.5%) and single (75.8%), while 35.8% had finished high school. The mean age of the participants was 40.85 years (SD = 15.13) (Tables 1 and 2). Table 1 shows that the mean length of drug exposure and the length of institutionalization for individuals in the SUD group were 20.42 years and 114.47 days, respectively.

**Table 1:** Age profile, length of drug use, and length of institutionalization (in days) according to measures of central tendency and dispersion

Groups	Variables	n	Minimum Value	Maximum Value	Median	Mean	Standard Deviation
Drug addiction	Length of drug use	60	1	62	17.00	20.42	14.63
	Length of institutionalization (in days)	60	1	545	50.50	114.47	14.63
	Age	60	18	72	39.50	41.12	13.74
Control	Age	60	21	75	42.00	40.58	16.51

**Table 2:** Absolute and percentage distribution according to sociodemographic characteristics.

Variables	Drug addiction		Control individuals		Total sample	
	n	%	n	%	n	%
Ethnicity						
White	40	66.7	35	58.3	73	62.5
Black	13	21.7	9	15.0	22	18.3
Brown (mixed race)	7	11.7	16	26.7	23	19.2
Marital status						
Married	12	20.0	16	26.7	28	23.3
Not married	47	78.3	44	73.3	91	75.8
Not Informed	1	1.7	-	-	1	0.8
Education						
Incomplete Elementary School	16	26.7	7	11.7	23	19.2
Complete Elementary School	3	5.0	4	6.7	7	5.8
Incomplete High School	13	21.7	4	6.7	17	14.2
Complete High School	20	33.3	23	38.3	43	35.8
Incomplete Higher Education	8	13.3	7	11.7	15	12.5
Complete Higher Education	-	-	15	25.0	15	12.5

The analysis of oral conditions revealed statistically significant differences between the variables associated with dental prosthesis (p < 0.001) and dental erosion (p < 0.001), indicating the worst conditions in the SUD group (Table 3). Table 4 shows statistically significant differences in the components of the DMFT index between the groups, indicating higher indices in the SUD group compared to the control group. Statistically significant differences in CPI were found in the healthy sextant (p < 0.001), — the highest values were observed in the control group —; in the sextant with calculus (p = 0.010); and in the sextant with periodontal pocket (p < 0.001) — the highest values were observed in the SUD group. As for CAL, statistically significant associations were found in the sextants with attachment loss ranging from 0 mm to 3 mm (p < 0.001), with the highest values in the control group; and in sextants with attachment loss of 12 mm or more (p = 0.036) in the SUD group (Table 4). Thus, individuals with substance use disorder showed the highest DMFT indices but the worst periodontal conditions (Table 3).

**Table 3:** Bivariate analysis between oral conditions (use of dental prosthesis; need for dental prosthesis; DMFT index; dental erosion) and study groups.

Variables	Drug addiction		Control individuals		p-value
	n	%	n	%	
Use of dental prosthesis					0.609*
No	52	86.7	50	83.3	
Yes	8	13.3	10	16.7	
Need for dental prosthesis					< 0.001*
No	24	40.0	55	91.7	
Yes	36	60.0	5	8.3	
DMFT index					0.126*
Below the index	35	58.3	43	71.7	
Above the index	25	41.7	17	28.3	
Dental erosion					< 0.001***
Erosion-free	23	39.7	48	84.2	
With erosion	35	60.3	9	15.8	

* Chi-square test

*** Likelihood ratio Test

DMTF index - below the index/above the index - mean of decayed teeth, missing, and filled teeth according to the values found in the studied groups.

**Table 4:** Comparative analysis between the components of the DMFT index, CPI, and CAL per sextant and study group.

Index		Groups	n	Median	Mean	Standard Deviation	p-value
DMFT	Decayed	Drug addiction	60	3.00	4.52	4.71	< 0.001**
		Control	60	0.00	0.68	1.32	
	Missing	Drug addiction	60	4.00	7.42	8.72	< 0.001**
		Control	60	0.00	3.22	7.55	
	Filled	Drug addiction	60	4.00	4.38	3.66	0.005**
		Control	60	1.00	2.63	3.17	
CPI	CPI: Healthy sextant	Drug addiction	59	0.00	1.00	1.84	< 0.001**
		Control	57	4.00	3.63	2.01	
	CPI: Sextant with bleeding	Drug addiction	59	0.00	1.17	1.65	0.232**
		Control	57	1.00	1.35	1.41	
	CPI: Sextant with calculus	Drug addiction	59	0.00	1.27	1.79	0.010**
		Control	57	0.00	0.44	0.78	
	CPI: Sextant with periodontal pocket	Drug addiction	59	1.00	1.34	1.72	< 0.001**
		Control	57	0.00	0.11	0.59	
CAL	Sextant with attachment loss 0-3	Drug addiction	59	1.00	1.81	2.26	< 0.001**
		Control	57	5.00	3.88	2.13	
	Sextant with attachment loss 4-5	Drug addiction	58	0.50	1.53	2.03	0.834**
		Control	57	1.00	1.32	1.49	
	Sextant with attachment loss 6-8	Drug addiction	59	0.00	0.54	1.29	0.193**
		Control	57	0.00	0.32	0.98	
	Sextant with attachment loss 9-11	Drug addiction	59	0.00	0.71	1.52	-
		Control	57	0.00	0.00	0.00	
	Sextant with attachment loss 12+ mm	Drug addiction	59	0.00	0.20	0.66	0.036**
		Control	57	0.00	0.07	0.53	

** Mann-Whitney U test

DMFT- Decayed, missing due to caries, and filled teeth index; CPI- Community periodontal index; CAL- Clinical attachment loss index.

Table 5 presents the salivary biochemical parameters and shows statistically significant differences in the SUD group than in the control group. Individuals in the control group also showed higher FRAP-values than those in the SUD group (Table 5).

**Table 5:** Comparative analysis between biochemical markers (cortisol, ferric reducing antioxidant power, thiobarbituric acid reactive substances, and carbonyl protein) and study group.

Markers	Groups	n	Median	Mean	Standard Deviation	p-value
Cortisol	Drug addiction	60	33.95	37.42	16.23	< 0.001*
	Control	60	24.65	26.05	13.57	
FRAP	Drug addiction	60	367,80	403,37	235,02	0.014**
	Control	60	428.00	460.41	162.86	
TBARS	Drug addiction	60	0.46	0.54	0.33	0.517**
	Control	60	0.51	0.59	0.41	
Carbonyl protein	Drug addiction	60	35.63	41.37	22.80	0.811**
	Control	60	37.22	43.60	24.14	

* T-test for means

** Mann-Whitney U test

FRAP- Ferric reducing antioxidant power; TBARS- Thiobarbituric acid reactive substances.

Table 6A shows the results for the SUD group. A weak but significant positive correlation (r = 0.291) was found between uric acid and CPI: sextant with bleeding, suggesting that an increase in uric acid is associated with an increase in CPI as well (p-value = 0.024). A moderate and statistically significant positive correlation (r = 0.390) was also observed between salivary amylase and DMFT: decayed teeth (p-value = 0.002) (Table 6A).

**Table 6A.** Correlation between biochemical markers (calcium, phosphorus, uric acid, amylase, total protein, and cortisol) and the study variables in the group of individuals with substance use disorder.

Variables	Calcium		Phosphorus		Uric acid		Amylase		Total protein			Cortisol	
Correlation coefficient	p-value	Correlation coefficient	p-value	Correlation coefficient	p-value	Correlation coefficient	p-value		Correlation coefficient	p-value	Correlation coefficient	p-value
DMFT: Decayed	0.156	0.233	0.236	0.069	-0.100	0.446	0.390*	0.002		0.094	0.474	0.158	0.228
DMFT: Missing	0.091	0.488	0.009	0.943	-0.031	0.815	0.238	0.067		0.091	0.491	0.263*	0.042
DMFT: Filled	-0.044	0.740	-0.207	0.112	0.145	0.269	0.003	0.984		-0.152	0.245	-0.061	0.641
DMFT: DMFT index	0.065	0.619	-0.010	0.942	-0.099	0.453	0.213	0.102		0.086	0.516	0.260*	0.045
CPI: Healthy sextant	0.223	0.087	-0.127	0.332	0.321	0.102	-0.162	0.215		0.009	0.944	-0.001	0.995
CPI: Sextant with bleeding	0.094	0.475	-0.071	0.590	0.291*	0.024	0.016	0.905		-0.097	0.462	-0.120	0.359
CPI: Sextant with calculus	-0.193	0.139	0.091	0.492	-0.184	0.159	-0.090	0.494		-0.074	0.576	-0.009	0.947
CPI: Sextant with periodontal pocket	-0.005	0.972	0.050	0.706	-0.203	0.120	0.107	0.415		0.218	0.094	-0.052	0.692
CPI: Excluded sextant	0.016	0.903	-0.026	0.846	-0.128	0.331	0.158	0.227		-0.035	0.788	0.160	0.221
CAL: loss 0-3mm	0.027	0.841	-0.148	0.259	0.039	0.769	-0.232	0.075		-0.185	0.157	-0.048	0.714
CAL: loss 4-5mm	0.083	0.530	0.116	0.379	0.248	0.056	0.042	0.748		0.012	0.925	0.078	0.553
CAL: loss 6+ mm	-0.105	0.425	0.058	0.657	-0.167	0.202	0.102	0.438		0.172	0.189	-0.208	0.111
CAL: Excluded sextant	0.016	0.903	-0.026	0.846	-0.128	0.331	0.158	0.227		-0.035	0.788	0.160	0.221

*Statistically significant Spearman’s correlation coefficient

DMFT- Decayed, missing due to caries, and filled teeth index; CPI- Community periodontal index; CAL- Clinical attachment loss index.

The SUD group showed weak and positive statistical correlations between cortisol level and the DMFT index (r = 0.260) (p-value = 0.045), and between cortisol level and the DMFT: missing teeth (r = 0.263) (p-value = 0.042) (Table 6A).

**Table 6B:** Correlation between biochemical markers (thiobarbituric acid reactive substances, carbonyl protein, ferric reducing antioxidant power, salivary flow, and salivary pH) and the study variables in the group of individuals with substance use disorder.

Variables	TBARS		Carbonyl protein		FRAP		Salivary flow		Salivary pH	
	Correlation coefficient	p-value	Correlation coefficient	p-value	Correlation coefficient	p-value	Correlation coefficient	p-value	Correlation coefficient	p-value
DMFT: Decayed	0.135	0.305	0.211	0.105	0.040	0.759	0.105	0.423	0.108	0.413
DMFT: Missing	0.254	0.051	0.079	0.548	0.026	0.842	-0.022	0.870	0.002	0.989
DMFT: Filled	0.012	0.928	-0.024	0.854	-0.080	0.545	0.022	0.866	0.025	0.849
DMFT: DMFT index	0.251	0.053	0.117	0.372	-0.053	0.686	0.040	0.762	0.048	0.714
CPI: Healthy sextant	-0.197	0.132	-0.154	0.241	0.003	0.981	-0.060	0.649	-0.230	0.077
CPI: Sextant with bleeding	-0.109	0.407	-0.054	0.682	-0.030	0.820	-0.143	0.275	-0.030	0.822
CPI: Sextant with calculus	0.103	0.434	0.068	0.606	0.051	0.696	0.040	0.762	0.070	0.595
CPI: Sextant with periodontal pocket	0.146	0.265	-0.045	0.732	0.055	0.676	-0.015	0.912	-0.039	0.769
CPI: Excluded sextant	0.185	0.158	0.167	0.202	-0.073	0.580	0.102	0.436	-0.037	0.782
CAL: loss 0-3mm	-0.029	0.825	0.098	0.454	0.090	0.495	-0.035	0.789	-0.071	0.588
CAL: loss 4-5mm	-0.187	0.153	-0.061	0.642	-0.135	0.304	-0.051	0.700	0.099	0.450
CAL: loss 6+ mm	0.074	0.575	-0.048	0.713	0.073	0.578	0.007	0.959	-0.072	0.585
CAL: Excluded sextant	0.185	0.158	0.167	0.202	-0.073	0.580	0.102	0.436	-0.037	0.782

*Statistically significant Spearman’s correlation coefficient

FRAP- Ferric reducing antioxidant power; TBARS- Thiobarbituric acid reactive substances.

DMFT- Decayed, missing due to caries, and filled teeth index; CPI- Community periodontal index; CAL- Clinical attachment loss index.

Table 7A presents the results for the control group. Statistically significant associations were found between amylase and the DMFT: missing teeth (r = 0.282) and between amylase and CAL: loss of 6 mm or more (r = 0.259) and CPI: excluded sextant (r = 0281). As salivary amylase increased, DMFT: missing teeth (p-value = 0.029), CAL: loss of 6 mm or more (p-value = 0.046), and CPI: excluded sextant (p-value = 0.030) also increased. A moderate negative correlation was observed between salivary amylase and CPI: healthy sextant (r = -0.352; p-value = 0.006) and CAL: loss between 0 mm and 3 mm (r = -0.437; p < 0.001) (Table 7A).

**Table 7A.** Correlation between biochemical markers (calcium, phosphorus, uric acid, amylase, total protein, and cortisol) and study variables in the control group.

Variables	Calcium		Phosphorus		Uric acid		Amylase		Total protein		Cortisol	
	Correlation coefficient	p-value	Correlation coefficient	p-value	Correlation coefficient	p-value	Correlation coefficient	p-value	Correlation coefficient	p-value	Correlation coefficient	p-value
DMFT: Decayed	-0.049	0.710	-0.138	0.293	0.095	0.469	0.210	0.107	-0.042	0.752	-0.091	0.490
DMFT: Missing	0.110	0.403	0.130	0.323	0.110	0.403	0.282*	0.029	0.161	0.219	0.277	0.085
DMFT: Filled	0.212	0.104	0.039	0.767	-0.004	0.976	-0.026	0.844	0.094	0.476	0.240	0.441
DMFT: DMFT index	0.204	0.119	0.247	0.057	0.097	0.462	0.236	0.070	0.261*	0.044	0.320	0.252
CPI: Healthy sextant	-0.031	0.815	-0.247	0.057	-0.236	0.070	-0.352*	0.006	-0.383*	0.003	0.240	0.221
CPI: Sextant with bleeding	0.027	0.836	0.044	0.739	0.291*	0.024	0.160	0.222	0.192	0.141	0.245	0.114
CPI: Sextant with calculus	-0.310*	0.016	0.116	0.376	-0.138	0.295	0.063	0.631	0.140	0.287	0.243	0.062
CPI: Sextant with periodontal pocket	0.018	0.890	-0.099	0.453	0.004	0.974	0.227	0.082	-0.219	0.092	0.061	0.641
CPI: Excluded sextant	0.210	0.107	0.147	0.263	0.160	0.223	0.281*	0.030	-0.274*	0.034	-0.223	0.086
CAL: loss 0-3mm	-0.015	0.911	-0.334*	0.009	-0.348*	0.007	-0.437*	0.000	-0.359*	0.005	0.206	0.114
CAL: loss 4-5mm	-0.025	0.850	0.059	0.655	0.379*	0.003	0.205	0.117	0.164	0.211	-0.101	0.441
CAL: loss 6+ mm	-0.133	0.309	0.183	0.161	-0.072	0.587	0.259*	0.046	0.109	0.408	0.156	0.234
CAL: Excluded sextant	0.198	0.129	0.195	0.134	0.127	0.335	0.248	0.056	0.279*	0.031	-0.161	0.221

*Statistically significant Spearman’s correlation coefficient

DMFT- Decayed, missing due to caries, and filled teeth index; CPI- Community periodontal index; CAL- Clinical attachment loss index.

We found other moderate and negative correlations in the control group, such as between calcium and CPI: sextant with calculus (r = -0.310), showing that as the concentration of calcium increased, the number of sextants with calculus decreased (p-value = 0.016). The same occurred with phosphorus concentration in the saliva and attachment loss from 0 to 3 mm (r = -0.334; p-value = 0.009) (Table 7A).

In addition, statistically significant associations were found between uric acid and CPI: sextant with bleeding (r = 0.291; p-value = 0.024), as well as associations between uric acid and CAL: loss between 4 mm and 5 mm (r = 0.379; p-value = 0.003); and CAL: loss between 0 mm and 3 mm (r = -0.348; p-value = 0.007).

Weak positive correlations were detected between TP and the DMFT index (r = 0.261; p-value = 0.044), CPI: excluded sextant (r = 0.274. p-value = 0.004), and CAL: excluded sextant (r = 0.279; p-value = 0.031), showing that as the TP concentration increased, so did the DMFT index and the number of excluded sextants. Other moderate and negative correlations were noted between TP, CAL: healthy sextant (r = -0.382; p-value = 0.003), and CAL: loss between 0 mm and 3 mm (r = -0.359; p-value = 0.005) (Table 7A).

Table 7B shows a statistically significant moderate negative correlation (r = -0.340) between salivary flow and the DMFT index, indicating that the DMFT index decreased (p-value = 0.008) as salivary flow increased. Moderate positive correlations (r = 0.377) were found between salivary flow and CPI: healthy sextant (p-value = 0.003) and CAL: loss between 0 mm and 3 mm (r = 0.352; p-value = 0.006). Salivary flow was also weakly and negatively correlated (r = -0.282) with CAL: loss of 6 mm or more, indicating that as salivary flow increased, attachment loss greater than 6 mm decreased (p-value = 0.029).

**Table 7B.** Correlation between biochemical markers (thiobarbituric acid reactive substances, carbonyl protein, ferric reducing antioxidant power, salivary flow, and salivary pH) and study variables in the control group.

Variables	TBARS		Carbonyl protein		FRAP		Salivary flow		Salivary pH	
	Correlation coefficient	p-value	Correlation coefficient	p-value	Correlation coefficient	p-value	Correlation coefficient	p-value	Correlation coefficient	p-value
DMFT: Decayed	-0.128	0.331	0.625	0.055	-0.130	0.323	-0.012	0.929	0.377	0.085
DMFT: Missing	-0.167	0.202	-0.239	0.066	-0.101	0.442	-0.226	0.083	0.277	0.085
DMFT: Filled	0.129	0.325	0.016	0.903	0.018	0.892	-0.237	0.068	0.240	0.441
DMFT: DMFT index	-0.095	0.471	0.521	0.040	-0.064	0.626	-0.340*	0.008	0.320	0.252
CPI: Healthy sextant	-0.011	0.935	0.210	0.050	0.290*	0.025	0.377*	0.003	0.240	0.221
CPI: Sextant with bleeding	0.207	0.112	-0.007	0.961	-0.234	0.072	-0.199	0.128	-0.091	0.490
CPI: Sextant with calculus	-0.011	0.931	0.012	0.928	-0.161	0.218	-0.035	0.791	0.277	0.085
CPI: Sextant with periodontal pocket	-0.029	0.824	-0.234	0.072	-0.241	0.064	-0.177	0.175	0.379	0.245
CPI: Excluded sextant	-0.166	0.206	0.208	0.113	-0.115	0.382	-0.223	0.087	0.277	0.085
CAL: loss 0-3 mm	0.044	0.736	0.101	0.443	0.279*	0.031	0.352*	0.006	0.240	0.441
CAL: loss 4-5 mm	0.006	0.961	-0.048	0.717	-0.169	0.196	-0.136	0.300	0.320	0.252
CAL: loss 6+ mm	0.068	0.604	-0.169	0.037	-0.318*	0.013	-0.282*	0.029	0.240	0.221
CAL: Excluded sextant	-0.175	0.182	0.052	0.420	-0.117	0.375	-0.173	0.187	-0.091	0.490

FRAP- Ferric reducing antioxidant power; TBARS- Thiobarbituric acid reactive substances.

DMFT- Decayed, missing due to caries, and filled teeth index; CPI- Community periodontal index; CAL- Clinical attachment loss index.

A weak positive correlation was observed between FRAP and CPI: healthy sextant (r = 0.290) and CAL: loss between 0 mm and 3 mm (r = 0.279), highlighting that as FRAP increased, the number of healthy sextants (p-value = 0.025) and the 0-3 mm attachment loss increased as well (p-value = 0.006). A moderate negative correlation was found between this parameter and CAL: loss of 6 mm or more (r = -0.318; p-value = 0.013).

## Discussion

The present study investigated clinical and laboratory parameters related to oral conditions in institutionalized individuals with and without substance use disorder, revealing various adverse outcomes associated with drug addiction and the impact of negative oral conditions on salivary parameters. In this investigation, specific biomarkers were considered, which were essential for the diagnosis, monitoring, and progression of the most prevalent oral conditions in individuals with drug addiction. Furthermore, these biomarkers are important resources for assessing salivary conditions with physiological and pathological implications, especially considering their origin, composition, functions, and interactions with organic systems.^18^ The use of illicit substances directly and indirectly affects oral health due to the deleterious effects and behavioral changes caused by drug abuse.^18^ We found that the SUD group had a larger number of tooth elements affected by dental erosion than did the control group. Mateos-Moreno et al.^19^ and Melo et al.^20^ found similar results. They pointed out that drug addiction, associated with the use of medicines, directly affects systemic health and decreases salivary flow, harming dental tissues and the oral mucosa. Another factor that can contribute to dental erosion in the SUD group is the consumption of carbonated drinks, such as alcohol.^21^

Dental caries and periodontal diseases are more prevalent in psychoactive substance users than in the general population.^22^ Ye et al.^23^ and Gupta^24^ used the DMFT index to show an increase in the average number of decayed, missing, and filled teeth in this population. This was consistent with our findings, which demonstrated that the SUD group had a higher mean number of decayed, missing, and filled teeth than those in the control group. Drug toxicity and length of use are factors that predispose to the risk of diseases, increase the DMFT index, and cause numerous tooth losses, leading to a greater need for dental prostheses, as observed in the SUD group.^22^

Although CPI considers calculus as an indicator of periodontal disease and does not differentiate between gingivitis and periodontitis as distinct oral problems, in this investigation, as in other national and international studies, the WHO criteria were adopted for evaluating general oral health, bleeding, presence of calculus, and periodontal pockets.^17^

The SUD group had the worst periodontal conditions, with the highest number of sextants with calculus, periodontal pockets, and attachment loss of 12 mm or more. Conversely, the control group had a larger number of healthy sextants and attachment loss between 0 mm and 3 mm. Gupta^24^ found similar results and showed that poor periodontal condition is a consequence of the lack of oral self-care among chemical substance users.^24^ Parvaei et al.^25^ associated the main cause of periodontal disease in this population with reduced salivary flow and reduced interest in maintaining oral health, resulting in an altered microbial profile.^25^

As a diagnostic tool, saliva has been widely used to map out and monitor the development and progression of oral health problems and stress.^25,26^ Salivary cortisol is an important hormone in modulating one’s response to physical and psychosocial stressors. When repeatedly exposed to these factors, one can have excessive cortisol hormone secretion and adverse health effects.^15^ In this study, the average cortisol level was higher in individuals with drug addiction. Other studies presented similar results, reporting that increased cortisol levels can be influenced by stressful situations, negative events throughout one’s life, use of chemical substances, and smoking habits.^15,17^ There were statistically significant associations between cortisol levels and the increase in the DMFT index and DMFT: missing teeth in the SUD group. Andrade et al.^28^and Tikhonova et al.^29^ proposed associations between changes in cortisol levels and a greater predisposition to dental caries, taking into account that stress can cause changes in salivary flow and composition, altering the oral microbiome and leading to greater accumulation of biofilm on tooth surfaces.^28,29^

In the SUD group, correlations were found between salivary amylase and the DMFT: decayed teeth, while in the control group, other associations could be noted between salivary amylase and oral conditions, such as DMFT: missing teeth, CPI: excluded sextant, and CAL: loss of 6 mm or more. Salivary amylase is an enzyme found abundantly in the acquired enamel film, capable of modulating bacterial colonization and providing the necessary substrates for biofilm formation.^29^ Rai et al.^18^ described a positive relationship between salivary amylase and the number of missing teeth, which is due to altered immune responses that contribute to increased bacterial colonization and the breakdown of periodontal attachment.^18,30^ The same conditions were observed by Parlak et al.,^31^ who found higher salivary amylase activity in patients with gingivitis and periodontitis. This condition can be explained by the fact that the high salivary amylase activity may be associated with the oral defense mechanism against inflammation, the stage of periodontal diseases, oral microbiome, nutritional habits, and salivary flow rate.^31^

The control group presented moderate and positive correlations between salivary flow, CPI: healthy sextant, and CAL: loss between 0 mm and 3 mm, emphasizing the bidirectional relationship between the salivary flow rate and periodontal health. There was also a moderate negative correlation between salivary flow and decreased DMFT index. According to Pedersen and Belstrøm,^32^ a normal stimulated or unstimulated salivary flow is essential for the fluid to fulfill its various functions, such as continuous lubrication, prevention of retrograde infections of the salivary glands, mechanical cleaning of the oral cavity, and dissolution of food debris and microorganisms, protecting the oral mucous membranes and tooth elements.^32^

Another correlation found in the control group refers to the salivary concentrations of calcium and phosphorus with CPI: sextant with calculus and AL: loss between 0 mm and 3 mm, respectively. As calcium and phosphorus concentrations increased, the number of sextants with calculus and attachment loss from 0 mm to 3 mm decreased. These outcomes corroborate the findings by Yaghini et al.,^33^ who found significant differences between calcium and phosphorus concentrations in patients with periodontitis. This phenomenon can be explained by the fact that high calcium and phosphorus levels in the saliva of patients diagnosed with periodontal diseases are common and occur as a result of the extravasation of these ions by the crevicular fluid.^33^

Uric acid is a major antioxidant in salivary composition, and its increased concentration is clinically important for monitoring oxidative stress.^34^ While this study found correlations between uric acid, CPI: sextant with bleeding, and CAL: loss between 4 mm and 5 mm in the control group, there was also a weak positive correlation in the SUD group between uric acid and CPI: sextant with bleeding. Our findings are in line with those of previous studies that showed significant differences between uric acid levels in healthy individuals and patients with periodontitis. This implies that uric acid not only induces an inflammatory response but that it is also associated with an increase in local and systemic oxidative stress, compromising antioxidant capacity.^35,36^

This study found that the control group had the highest FRAP-values compared to the SUD group. In addition, correlations were observed between FRAP and CPI: healthy sextant, CAL: loss between 0 mm and 3 mm, and CAL: loss of 6 mm or more. Becerik et al.^37^ found a similar result. They observed that FRAP-values were higher in the group with a healthy periodontal status. Given that FRAP is an antioxidant defense system in one’s body, it prevents the accumulation of free radicals to reduce the oxidative imbalance caused by periodontal disease.

Thus, diagnosing oral conditions in individuals with drug addiction and treating the deleterious effects of drug abuse are important to restore self-esteem and improve individuals’ quality of life. Furthermore, in this context, dentistry is an inductive tool for promoting general and oral health, helping individuals cope with drug addiction and assisting them in social reintegration.^9, 38^

## Study limitations

The design of this study is a possible limitation because, in cross-sectional studies, exposure and outcome are assessed simultaneously, making it difficult to establish a temporal relationship between them. The duration of monitoring of the SUD group was also a limitation.

## Conclusion

This study showed that individuals with drug addiction had worse oral conditions compared to control individuals, such as the need for prosthetic devices, an above-average DMFT index, a greater number of teeth affected by dental erosion, higher levels of attachment loss, and the presence of sextants with calculus and periodontal pockets. In addition, oral conditions influenced salivary biochemical parameters in both groups, with higher levels of cortisol, uric acid, and salivary amylase in the SUD group.
